# Symphysis morphology and mandibular alveolar bone thickness in patients with β-thalassemia major and different growth patterns

**DOI:** 10.1590/2177-6709.27.2.e22205.oar

**Published:** 2022-05-23

**Authors:** Leila KHOJASTEPOUR, Atefe NADERI, Fatemeh AKBARIZADEH, Najmeh MOVAHHEDIAN, Farzaneh AHRARI

**Affiliations:** 1 Shiraz University of Medical Sciences, School of Dentistry, Department of Oral and Maxillofacial Radiology (Shiraz, Iran).; 2 Mashhad University of Medical Sciences, School of Dentistry, Dental Research Center (Mashhad, Iran).

**Keywords:** Alveolar bone, Alveolar process, Mandible, Orthodontics, Symphysis, Thalassemia

## Abstract

**Objective:**

The present study aimed to assess the morphology of symphysis and alveolar bone thickness (ABT) surrounding mandibular incisors in thalassemic patients, as compared to unaffected individuals.

**Methods:**

This case-control study was conducted on lateral cephalograms of 60 thalassemic and 60 unaffected patients with Class II malocclusion seeking orthodontic treatment at Dental School, Shiraz University of Medical Sciences. The sample was divided into three subgroups including hyperdivergent, normodivergent, and hypodivergent, according to the Jarabak index. Symphysis dimensions and alveolar bone thickness surrounding mandibular incisors were measured using AutoCad software. Finally, the correlation between alveolar bone thickness and symphysis morphology was assessed.

**Results:**

In general, chin dimensions and bone thickness at different levels of mandibular incisor roots (cervical, middle, apical) were smaller in thalassemic adolescents than controls. Concerning the total sample as well as the normodivergent subgroup, significantly lower values were observed in thalassemic patients for symphysis width, total ABT at the cervical, and lingual ABT at the apical root area compared to controls (*p* < 0.05). The hypodivergent growth pattern was not associated with any statistical differences between the groups (*p*> 0.05). In both thalassemic and control subjects, symphysis width showed a weak to moderate positive correlation with ABT of lower incisors (*p*< 0.05), whereas symphysis height showed a moderate positive correlation with cervical ABT in only ß‐thalassemia patients (*p*< 0.05).

**Conclusions:**

Compared to controls, ß-thalassemia patients showed thinner alveolar bone at different levels of lower incisor roots and smaller symphysis dimensions. There were significant correlations between symphysis dimensions and alveolar bone thickness of mandibular incisors in the sample.

## RESUMO

**Objetivo:**

O presente estudo teve como objetivo avaliar a morfologia da sínfise e a espessura do osso alveolar (EOA) ao redor dos incisivos inferiores de pacientes com talassemia, em comparação a indivíduos sem a doença.

**Métodos:**

Esse estudo de caso-controle foi conduzido por meio da análise de radiografias cefalométricas de pacientes Classe II à procura de tratamento ortodôntico na Clínica Odontológica da *Shiraz University of Medical Sciences*, sendo 60 pacientes com talassemia e 60 sem a doença. A amostra foi dividida em três subgrupos, de acordo com o índice de Jarabak: hiperdivergente, normodivergente e hipodivergente. As dimensões da sínfise e a espessura do osso alveolar ao redor dos incisivos inferiores foram medidas no programa AutoCAD. Por último, foi avaliada a correlação entre a espessura do osso alveolar e a morfologia da sínfise.

**Resultados:**

No geral, as dimensões do mento e a espessura do osso nos diferentes níveis da raiz dos incisivos inferiores (cervical, médio e apical) foram menores em adolescentes talassêmicos do que nos pacientes controle. Tanto na amostra total quanto no subgrupo normodivergente, valores significativamente menores foram observados nos pacientes talassêmicos para a largura da sínfise, EOA total no terço cervical e EOA lingual no terço apical da raiz, comparados aos pacientes controle (*p*< 0,05). O padrão de crescimento hipodivergente não foi associado a qualquer diferença estatisticamente significativa entre os grupos (*p*> 0,05). Em ambos os grupos de pacientes, talassêmicos e controle, a largura da sínfise mostrou uma correlação positiva de fraca a moderada com a EOA dos incisivos inferiores (*p*< 0,05), enquanto a altura da sínfise mostrou uma correlação positiva moderada com a EOA cervical apenas nos pacientes com talassemia beta (*p*< 0,05).

**Conclusões:**

Comparados aos pacientes controle, os pacientes com talassemia beta apresentaram um osso alveolar mais fino em diferentes níveis das raízes dos incisivos inferiores e dimensões menores da sínfise. Houve correlação significativa entre as dimensões da sínfise e a espessura do osso alveolar dos incisivos inferiores na amostra.

## INTRODUCTION

Mandibular symphysis plays a key role in the balance of the face and beauty of the profile.^1^ ^,^ ^2^ Therefore, assessment of chin shape and size is of crucial importance in orthodontic and surgical treatment planning. Several studies demonstrated that the morphology and anatomical features of mandibular symphysis is influenced by the vertical and anteroposterior growth pattern as well as by sex and also the position and inclination of mandibular anterior teeth.^1^ ^,^ ^3^ ^-^ ^6^ It is known that vertical facial pattern has a strong influence on chin shape, so that in the brachyfacial pattern, the symphysis is usually short and wide, whereas in dolichofacial subjects, the symphysis is narrow and high.^4^ ^,^ ^7^ The position and inclination of mandibular anterior teeth also influence the morphology of the surrounding alveolar bone and in this way may affect chin morphology.^1^ ^,^ ^6^

Whether the morphology of symphysis is influenced by the position of mandibular incisors, there is no doubt that the thickness of symphysis limits the movement of mandibular anterior teeth during orthodontic treatment. As demonstrated by Proffit et al,^8^ tooth movement occurs within a boundary called the envelope of discrepancy. When teeth are moved beyond the anatomical limits of the surrounding alveolar bone, iatrogenic sequelae such as decreased alveolar bone thickness, dehiscence or fenestration of the buccal or lingual cortical plates and dental mobility may occur.^4^ ^,^ ^9^ ^,^ ^10^ Among the different parts of upper and lower jaws, the buccal and lingual alveolar bone in the mandibular incisor region is very thin, particularly at the upper root half, so that dehiscence and fenestration of alveolar bone is frequently observed in this area.^10^ ^,^ ^11^ Respecting tooth movement boundaries is especially important in patients with severe malocclusion, where there are usually natural dentoalveolar compensations that may be increased during orthodontic treatment.^12^ Accurate preoperative evaluation of mandibular symphysis is also beneficial for clinicians who decide to place implants in the mandibular anterior region.

Thalassemia is one of the most common genetic disorders across the world.^13^ It is found in more than 60 countries, with the highest distribution in the Mediterranean region, parts of Africa, the Middle East, the Indian subcontinent, Far East and South East Asia.^14^ ^,^ ^15^ Thalassemia can cause considerable changes in facial appearance of patients, due to retardation and alteration in the growth and development of bones.^14^ One important manifestation of thalassemia is bone marrow expansion, especially of the skull, which leads to facial dysmorphology.^14^ ^,^ ^16^ The appearance of the face in patients with beta-thalassemia major has been resembled to a “rodent face”.^17^ Improvements in the medical management of thalassemic patients have led to the increased life expectancy and willingness to seek esthetic and orthodontic treatments. 

Prior studies have shown that patients with β-thalassemia major tend to have prominent dentofacial characteristics, including Class II malocclusion with a protrusive premaxilla, posterior rotation of the mandible, short length of the mandibular body, flaring and spacing of the maxillary anterior teeth, increased overjet and reduced overbite, as compared to normal individuals.^14^ ^,^ ^15^ ^,^ ^18^ ^-^ ^20^ However, there is little research in the literature regarding chin morphology and bone thickness measurements around mandibular incisors in patients with β-thalassemia major. Thus, the present study aimed to measure the dimensions of symphysis and the thickness of alveolar bone at different root levels of mandibular anterior teeth in thalassemic patients with different vertical patterns, and compare the results with corresponding values in a group of unaffected individuals. The second aim of the study was to detect any correlation between symphysis morphology and alveolar bone thickness (ABT) of the mandibular anterior teeth in the sample.

## MATERIAL AND METHODS

### SUBJECTS

A sample of 60 lateral cephalograms was obtained from the files of patients with β-thalassemia major who sought orthodontic treatment at the Department of Orthodontics, School of Dentistry, Shiraz University of Medical Sciences (Shiraz, Iran), Ref. n. IR.SUMS.REC.1394.S1081. The sample size was calculated based on the significance level of 0.05 (α=0.05) and power of 80% (β=0.20) to detect a mean difference of 0.5 mm in bone thickness between the two groups, with a standard deviation of 0.90. The sample size was calculated as 51 and then increased to 60 per group, in order to increase the power of the study.

The cases were eligible if they were between 11 to 15 years age and showed Angle Class II malocclusion. The lateral cephalograms of patients who had any history of prior orthodontic treatment or showed missing lower anterior teeth, as well as those who reported any past trauma to the mandible or symphysis were excluded from the sample. The images with poor quality and clarity were also rejected.

The cephalograms of 60 healthy controls with Class II malocclusion, similar vertical facial pattern and similar chronological age range (± 6 months) and sex ratio were recruited from the pre-treatment records of the patients at the Orthodontic Department of the same center. The exclusion criteria for the control group were the same as that described for the case patients. The study protocol was approved by the Regional Bioethics Committee. 

### THE CEPHALOMETRIC ANALYSIS

Lateral cephalograms were taken at the natural head position with teeth in maximum occlusion and lips in repose using a Planmeca X-ray equipment (Planmeca ProMax^®^, Helsinki, Finland) with the following specifications: 2.5 mm Al total filtration, 0.5×0.5 mm focal spot size, 60-84 kV anode voltage, 1-16 mA anode current, and 0.2-5 seconds exposure time. The images were digitized using a table scanner (Microtek ScanMaker i800; Microtek International Inc., Carson, CA, USA) at a resolution of 300 dpi, and saved in bitmap format. The scanning process was performed in a 1:1 ratio. The cephalometric analysis was performed digitally by means of the AutoCAD 2007 software (Autodesk Inc., CA, USA). The identification of landmarks was made by two calibrated oral and maxillofacial radiologists, and the angular and linear measurements were recorded to the nearest 0.5° and 0.5 mm, respectively. To calculate the intraexaminer and interexamineres reliability, 10% of the cephalometric radiographs were randomly selected and all the measurements were repeated by both radiologists, one week later.

#### DETERMINATION OF VERTICAL GROWTH PATTERN

The Jarabak index was used to determine the vertical growth pattern (facial type) in β-thalassemic patients and controls. The Jarabak index was calculated by dividing the posterior facial height (S-Go; the distance between points Sella and Gonion) by the anterior facial height (N-Me; the distance between points Nasion and Menton). Accordingly, the lateral cephalograms were classified into three subgroups, as follows:

1. Hyperdivergent growth pattern (dolichofacial type), with Jarabak index < 62%.
2. Normodivergent growth pattern (mesofacial type), with Jarabak index of 62-65%.
3. Hypodivergent growth pattern (brachyfacial type), with Jarabak index > 65%.

#### DETERMINATION OF ALVEOLAR BONE THICKNESS (ABT) OF MANDIBULAR ANTERIOR TEETH AND ITS CORRELATION WITH SYMPHYSIS DIMENSIONS

**Figure 1:**
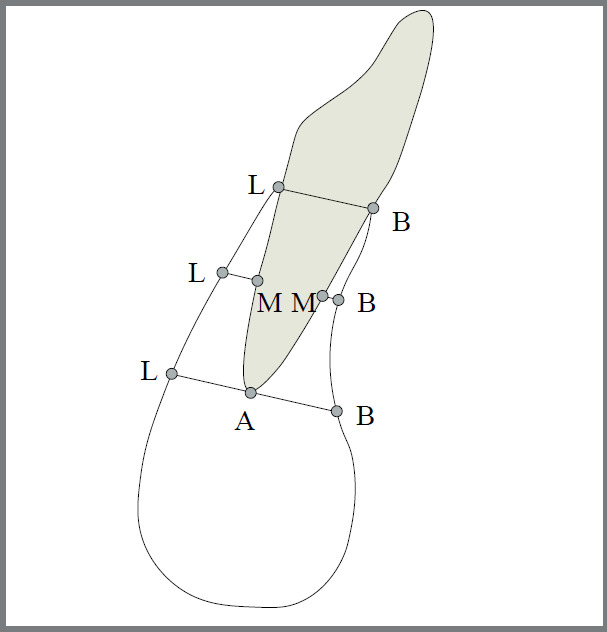
Tracing of the mandibular incisor and symphysis configuration. The alveolar bone thickness was measured at three levels (cervical, middle and apical).

**Figure 2:**
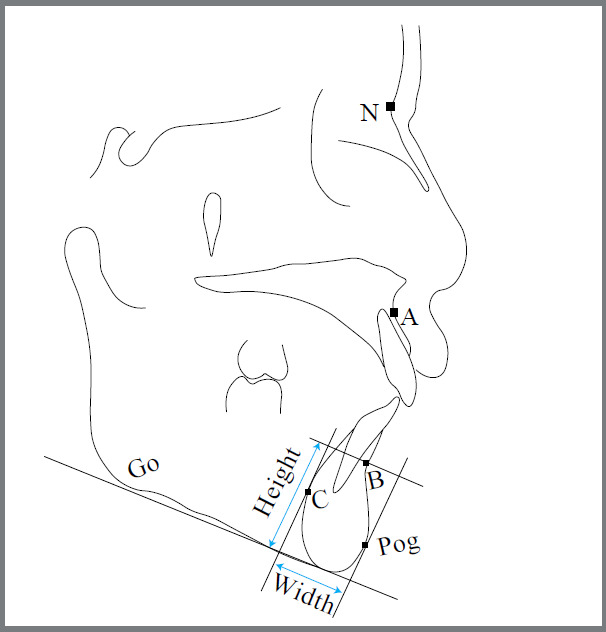
Tracing of the cephalogram pertaining to a boy with β-thalassemia major, to calculate symphysis width and height.

The configuration of mandibular symphysis was drawn on lateral cephalograms inside the special window of the software. After tracing the symphysis, nine cephalometric landmarks were identified, to yield five linear measurements for determining the alveolar bone thickness (ABT) surrounding mandibular incisors (Fig 1). Table 1 presents the definition of five cephalometric parameters for assessment of alveolar bone thickness. The symphysis height and width were also determined as illustrated in Figure 2. For this purpose, the most anterior point on symphysis (pogonion) as well as the most posterior point on the lingual aspect of symphysis (point C) were marked. Then a line was drawn from each of these points perpendicular to the mandibular plane. A line was also drawn from point B (the deepest point on the concavity of the mandibular profile between the alveolar crest and pogonion) parallel to the mandibular plane, so that a rectangle was formed. The length and width of this rectangle represented the symphysis height and width, respectively (Fig 2). All of the measurements were performed by the software. Finally, the correlation between alveolar bone thickness and symphysis dimensions was assessed. The magnification factor of 1.13 for the cephalograms was not adjusted, as all the images were taken by the same device.

**Table 1:** Linear variables for assessment of alveolar bone thickness around lower anterior teeth

Measurement	Description
B-L	Width of the alveolar process at the cervical region of mandibular incisors (at the level of the vestibular and lingual bone crests)
B-M	Buccal bone thickness at the midroot of the mandibular incisor. It was measured from half the root of the most labially mandibular incisor to the external limit of the buccal cortex of the mandibular symphysis.
L-M	Lingual bone thickness at the midroot of the mandibular incisor. It was measured from half the root of the most labially mandibular incisor to the external limit of the lingual cortex of the mandibular symphysis.
B-A	Buccal bone thickness at the apex of the mandibular incisor. It was measured from the apex of the most labially mandibular incisor to the external limit of the buccal cortex of the mandibular symphysis.
L-A	Lingual bone thickness at the apex of the mandibular incisor. It was measured from the apex of the most labially mandibular incisor to the external limit of the lingual cortex of the mandibular symphysis.

### STATISTICAL ANALYSIS

The Kolmogorov-Smirnov test confirmed that the data were normally distributed (*p*>0.05). The intraexaminer and interexaminers reliability was assessed by intraclass correlation coefficient (ICC). The difference in sex and age between the β-thalassemia and control groups was assessed by the chi-square test and student’s t-test, respectively. The Student’s *t*-test was also used to analyze the difference in symphysis dimensions and alveolar bone thickness variables between the patients with β-thalassemia major and unaffected controls. The Pearson correlation coefficient was calculated to determine the linear correlation between symphysis dimensions and alveolar bone thickness of the mandibular anterior teeth. The level of statistical significance was set at α = 0.05. The statistical analysis was performed using standard statistical software (SPSS, version 18.0; SPSS Inc., Chicago, Il, USA). 

## RESULTS

### SAMPLE CHARACTERISTICS

There were 34 females and 26 males with a mean age of 13.0 ± 1.6 years in the β-thalassemia group. The control subjects comprised 32 females and 28 males, with a mean age of 13.1 ± 1.7 years. The age (*p*= 0.580) and gender (*p*= 0.714) distribution was comparable between the two groups. The intraclass correlation coefficients ranged from 91.2% to 94.9%, showing excellent intraexaminer and interexaminers reliability. 

According to the Jarabak index (the proportion of total posterior facial height [S-Go] to total anterior facial height [N-Me]), the case group consisted of 32 hyperdivergent, 15 normodivergent, and 13 hypodivergent individuals. The mean Jarabak indices of the three subgroups were 57.97 ± 0.03, 62.80 ± 0.01 and 67.30 ± 0.02 percent, respectively. On the other hand, there were 33 hyperdivergent, 14 normodivergent, and 13 hypodivergent subjects in the control group with the mean Jarabak indices of 58.43 ± 0.04, 63.47 ± 0.01, and 69.70 ± 0.04 percent, respectively. 

### COMPARISON OF SYMPHYSIS DIMENSIONS AND ALVEOLAR BONE THICKNESS (ABT) AROUND MANDIBULAR INCISORS IN SUBJECTS WITH Β-THALASSEMIA MAJOR AND CONTROLS

Table 2 presents the descriptive data for the symphysis dimensions and alveolar bone thickness around lower incisors in β-thalassemia patients and controls, according to the vertical growth pattern. In general, the chin dimensions and alveolar bone thickness at different levels of mandibular incisor roots (cervical, middle, apical) were smaller in adolescents with β-thalassemia major than control subjects, although the difference between the groups was small and did not reach statistical significance in most areas. Concerning the total sample, the symphysis width and B-L and L-A parameters were significantly smaller in the thalassemic than the control group (*p*< 0.05, Table 2).

**Table 2:** Comparison of alveolar bone thickness around mandibular anterior teeth in β-thalassemia and unaffected adolescents, according to the vertical growth pattern.

Vertical pattern	Group	B-L	L-M	B-M	L-A	B-A	Symphysis width	Symphysis height
		Mean ± SD	Mean ± SD	Mean ± SD	Mean ± SD	Mean ± SD	Mean ± SD	Mean ± SD
Hyperdivergent	β-thalassemia	5.18 ± 0.59	1.15 ± 0.20	1.04 ± 0.21	2.99 ± 0.75	3.70 ± 1.12	1.15 ± 0.14	2.10 ± 0.27
	Control	5.20 ± 0.84	1.30 ± 0.29	1.10 ± 0.29	3.65 ± 0.71	3.98 ± 1.28	1.24 ± 0.17	2.14 ± 0.25
	*p*-value	0.889	0.019	0.305	0.001	0.346	0.579	0.884
Normodivergent	β-thalassemia	4.85 ± 0.53	1.30 ± 0.36	1.16 ± 0.31	3.19 ± 0.66	4.79 ± 1.49	1.27 ± 0.05	1.87 ± 0.25
	Control	5.72 ± 0.76	1.25 ± 0.25	1.31 ± 0.46	3.73 ± 0.68	4.87 ± 1.49	1.35 ± 0.14	2.13 ± 0.25
	*p*-value	0.001	0.698	0.330	0.040	0.882	0.03	0.984
Hypodivergent	β-thalassemia	5.17 ± 0.74	1.32 ± 0.31	1.06 ± 0.46	3.06 ± 1.15	3.79 ± 1.36	1.25 ± 0.11	2.10 ± 0.24
	Control	5.64 ± 0.79	1.40 ± 0.33	1.14 ± 0.21	3.39 ± 0.44	3.98 ± 1.28	1.28 ± 0.15	2.02 ± 0.33
	*p*-value	0.131	0.544	0.576	0.348	0.720	0.107	0.368
Total	β-thalassemia	5.1 ± 0.6	1.0 ± 0.3	1.2 ± 0.2	3.58 ± 0.82	3.9 ± 1.3	1.20 ± 0.13	2.05 ± 0.27
	Control	5.4 ± 0.8	1.1 ± 0.3	1.3 ± 0.2	3.61 ± 0.66	4.1 ± 1.3	1.28 ± 0.17	2.11 ± 0.27
	*p*-value	0.018	0.101	0.145	< 0.001	0.422	0.012	0.190

Data are shown as mean ± SD (standard deviation). B-L: Width of the alveolar process at the cervical region of mandibular incisors; L-M: Lingual bone thickness at the midroot of the mandibular incisor; B-M: Buccal bone thickness at the midroot of the mandibular incisor; L-A: Lingual bone thickness at the apex of the mandibular incisor; B-A: Buccal bone thickness at the apex of the mandibular incisor.

When the difference in symphysis dimensions and ABT measurements between the two groups was analyzed according to the vertical growth pattern, it was revealed that in the hyperdivergent subgroup, the mean values of L-M and L-A were significantly smaller in patients with β-thalassemia major than the control group (*p*< 0.05, Table 2). In normodivergent growth pattern, the symphysis width, and B-L and L-A measurements showed significantly lower values in the thalassemic than the control subjects (*p*< 0.05,Table 2). The hypodivergent growth pattern was not associated with a significant difference in chin morphology and ABT measurements of mandibular incisors between the groups (*p*> 0.05, Table 2).

### THE RELATIONSHIP BETWEEN ALVEOLAR BONE THICKNESS (ABT) OF LOWER INCISORS AND SYMPHYSIS MEASUREMENTS

Table 3 presents the relationship between ABT of mandibular incisors and symphysis width and height in the sample. In the β-thalassemia group, symphysis width showed a positive correlation with lingual (r = 0.306, *p* = 0.017) and buccal (r = 0.268, *p* = 0.039) ABT at the apical region, whereas symphysis height had just a positive correlation with total ABT at the cervical root area (r = 0.321, *p* = 0.013). 

**Table 3:** Correlations between mandibular symphysis dimensions and alveolar bone thickness of lower anterior teeth.

Group	Mandibular symphysis measurements		B-L	L-M	B-M	L-A	B-A
β-thalassemia	Symphysis width	Correlation Coefficient	0.054	-0.133	0.171	0.306	0.268
		*p*-value	0.681	0.310	0.190	0.017	0.039
	Symphysis height	Correlation Coefficient	0.321	-0.101	-0.033	-0.114	-0.179
		*p*-value	0.013	0.444	0.800	0.385	0.171
Control	Symphysis width	Correlation Coefficient	0.312	0.158	0.417	0.064	0.300
		*p*-value	0.015	0.228	0.001	0.628	0.020
	Symphysis height	Correlation Coefficient	0.184	-0.149	0.151	0.010	0.252
		*p*-value	0.160	0.257	0.249	0.942	0.052

Data were analyzed by Pearson’s correlation test. B-L: Width of the alveolar process at the cervical region of mandibular incisors; L-M: Lingual bone thickness at the midroot of the mandibular incisor; B-M: Buccal bone thickness at the midroot of the mandibular incisor; L-A: Lingual bone thickness at the apex of the mandibular incisor; B-A: Buccal bone thickness at the apex of the mandibular incisor.

In the control group, symphysis width showed a positive correlation with buccal ABT at the apical (r = 0.30, *p* = 0.02) and middle (r = 0.417, *p* = 0.001) root region and total ABT at the cervical (r = 0.312, *p* = 0.015) root area, but symphysis height had no significant correlation with alveolar bone thickness variables (*p* > 0.05; Table 3).

All significant correlations found between ABT of mandibular incisors and symphysis dimensions were weak to moderate (r = 0.27 to r = 0.42). 

## DISCUSSION

A comparative study was made between β-thalassemia and unaffected subjects with different vertical facial patterns, to determine symphysis dimensions and alveolar bone thickness at the apical, middle and cervical thirds of the roots of mandibular anterior teeth. According to the authors’ knowledge, this is the first study that made such an assessment in subjects with β-thalassemia major. The subjects were in the age range of 11 to 15 years, as this is the age range that patients usually refer for orthodontic treatment. Although most studies concerned about bone thickness at the root apex level of mandibular central incisors, the measurements at the cervical and middle thirds of the roots are also important. It has been demonstrated that buccal alveolar bone at the upper root half of mandibular incisors is thin in many patients, and therefore the occurrence of dehiscence and fenestration is frequent at this area.^10^ This highlights the importance of measuring alveolar bone thickness at the middle and cervical thirds of the mandibular incisor roots. 

According to the outcomes of this study, patients with β-thalassemia major generally showed thinner buccal and lingual alveolar bone at different root levels, compared to unaffected subjects. However, the differences in bone thickness between groups did not reach statistical significance in most areas. Considering the total sample as well as the normodivergent subgroup, the alveolar process at the cervical region of the tooth (B-L) and the lingual bone at the apex of the lower incisors (L-A) was significantly thinner in thalassemic than unaffected subjects. In hyperdivergent subgroup, the mean values of lingual ABT at the middle and apical levels of lower anterior teeth (L-M and L-A) were significantly smaller in patients with β-thalassemia major than the control group. It should be noted that the alveolar bone in the mandibular anterior region is thin even in unaffected subjects.^10^ ^,^ ^11^ Several factors such as the eccentric position or severe inclination of mandibular incisors, anterior crowding, as well as labial orthodontic tooth movement may aggravate the condition and lead to gingival recession, bony dehiscence and fenestration in the mandibular anterior area either before or during orthodontic treatment.^21^ ^-^ ^23^ The outcomes of this study indicate that thalassemic subjects are even at a greater risk of gingival and alveolar bone loss due to inherently thinner alveolar bone in the mandibular incisor area. Furthermore, Class II malocclusion is a very common condition in thalassemic patients, and is usually manifested by retroclined or upright maxillary incisors and proclined mandibular incisors. When mandibular teeth are more proclined, the buccal alveolar bone tends to be thinner and more susceptible to resorption during orthodontic treatment. Therefore, to compensate arch length discrepancy in thalassemic patients, the extraction approach may be preferred instead of buccal tooth movement, in order to prevent iatrogenic damage in the symphysis area.

Regarding symphysis dimensions, the present study revealed that patients affected with β-thalassemia major generally have smaller symphysis width and height (i.e. narrower and shorter symphysis) compared to unaffected individuals. The difference between groups, however, was small and statistical significance was only found in symphysis width in the total thalassemic sample as well as the normodivergent subgroup, whereas the symphysis height was not significantly different between the groups. The mandibular symphysis is of considerable importance in orthodontic treatment planning, as it plays a great role in the esthetics of facial profile, and also limits orthodontic movements due to its dense cortical structure. The narrow symphysis in thalassemic subjects is consistent with their thinner alveolar bone in the incisor area. Several studies indicated that patients with narrow and long symphysis have less bone support than those with wide and short symphysis,^10^ ^,^ ^24^ which makes them susceptible to the loss of both buccal and lingual alveolar bone.^9^ ^,^ ^10^

In the present study, the hypodivergent growth pattern was not associated with any significant difference in alveolar bone thickness and chin morphology between thalassemic and control subjects. This may be related to the small sample size in the hypodivergent subgroup. Furthermore, short face subjects generally show more retroclined incisors, compared to normal face and long face subjects, which can lead to thicker buccal cortical bone due to alveolar bone remodeling.^25^ Therefore, labial orthodontic tooth movement may be performed with lower risk of damage to periodontal tissues in hypodivergent thalassemic subjects. 

The present study demonstrated some interactions between symphysis dimensions and alveolar bone thickness (ABT) of mandibular incisors. Based on the results of the present study, symphysis width showed a weak to moderate positive correlation with lingual and buccal ABT at the apical region (L-A and B-A) in the β-thalassemia group, whereas in the control group, symphysis width showed a weak to moderate positive correlation with buccal ABT at the apical (B-A) and middle (B-M) root region and total ABT at the cervical root area (B-L). This means that in general, the wider the symphysis, the thicker the alveolar bone tended to be in the apical root region of thalassemic patients and in the whole incisor root length of unaffected subjects. 

In this study, symphysis height showed a moderate positive relationship with width of the alveolar process at the cervical root region (B-L) of thalassemic patients, but there was no significant correlation between symphysis height and any of the ABT measurements in unaffected individuals. The findings of this study indicate that in thalassemic subjects, the longer the symphysis, the thicker the alveolar bone would be at the cervical root region of mandibular incisors. It should be noted that all the correlations observed between the symphysis dimensions and ABT of mandibular incisors in the present investigation were weak to moderate, as the correlation coefficients ranged from 0.268 to 0.417. This means that symphysis dimensions could not be used as a strong measure for predicting the alveolar bone support around mandibular incisors.

So far, no study has compared the correlation of symphysis morphology with alveolar bone thickness of mandibular incisors in patients suffering from β-thalassemia major. Therefore, comparison of the results of this study with other investigations is limited. Some studies performed on craniofacial features of thalassemic patients evaluated cranial base and maxillary and mandibular parameters of thalassemic children, and demonstrated that patients with β-thalassemia major usually manifest a Class II skeletal relationship associated with a pronounced vertical growth pattern (skeletal open bite), small and retrognathic mandible, short ramus, a more convex facial profile, reduced Saddle angle and short posterior cranial base.^14^ ^,^ ^15^ ^,^ ^18^ ^-^ ^20^ ^,^ ^26^ In the present study, the percentage of hyperdivergent growth pattern was greater than other facial types in the sample. This finding is in agreement with the results of previous studies^14^ ^,^ ^15^ showing that vertical growth pattern is the more prevalent facial type among thalassemic subjects.

The present study indicated some differences in ABT of mandibular incisors and chin shape between thalassemic and control subjects. Although these differences were relatively small, they provide useful data for the clinician concerning the symphysis morphology and orthodontic treatment planning in Class II thalassemic subjects. The magnification factor of 1.13 for the X-ray machine was the same in all the cephalograms and could not affect the results, although the actual values would be a bit lower than that reported in the present investigation. The limitations of this study were the small sample size, especially in the hypodivergent subgroup, matching the groups according to the chronological age, and the use of lateral cephalometry for measuring the width and height of symphysis and ABT of mandibular incisors. Since the cephalograms had been taken with a thyroid collar, the second and third cervical vertebras were covered and we could not calculate the skeletal age and match the groups accordingly. The sample of this study was obtained from the available records of patients referred for orthodontic treatment, and it was not possible to make the measurements in three-dimensional (3D) cone-beam computed tomography (CBCT) scans. The use of CBCT images allows a more accurate measurement of symphysis dimensions and alveolar process thickness, and helps a reliable diagnosis of fenestration and dehiscence in this area.^27^ ^-^ ^29^ It is suggested that future studies use this advanced imaging technique on a larger sample of thalassemic patients, for three-dimensional analysis of the facial characteristics and symphysis morphology prior to orthodontic treatment and dental implant placement. It is also better to include non-growing patients or match the groups according to the skeletal age, in order to avoid the deviations arising from growth retardation in thalassemic, compared to control individuals. 

## CONCLUSION

1. In general, chin dimensions and alveolar bone thickness (ABT) at different thirds of mandibular incisor roots (cervical, middle, apical) were smaller in thalassemic patients than the control group.
2. In the total thalassemic sample as well as the normodivergent subgroup, the symphysis was significantly narrower and alveolar bone was significantly thinner at the cervical, and the lingual-apical area of mandibular incisor roots, as compared to controls.
3. The hypodivergent growth pattern was not associated with any significant difference in symphysis morphology and alveolar bone thickness between the two groups.
4. In both thalassemic and control subjects, symphysis width showed a weak to moderate positive correlation with ABT of mandibular incisors, whereas symphysis height showed a moderate positive correlation with cervical ABT in only β-thalassemia patients.
